# Analytical challenges encountered and the potential of supercritical fluid chromatography: A perspective of five experts

**DOI:** 10.1002/ansa.202000151

**Published:** 2020-12-01

**Authors:** Susan Olesik, Caroline West, Davy Guillarme, Debby Mangelings, Lucie Novakova

**Affiliations:** ^1^ Department of Chemistry and Biochemistry The Ohio State University Columbus Ohio USA; ^2^ Institut de Chimie Organique et Analytique University of Orleans Orleans France; ^3^ School of Pharmaceutical Sciences University of Geneva University of Lausanne Geneva Switzerland; ^4^ Department of Analytical Chemistry, Applied Chemometrics and Molecular Modelling Vrije Universiteit Brussel Brussels Belgium; ^5^ Department of Analytical Chemistry Faculty of Pharmacy, Charles University Hradec Králové Czech Republic

The first discussions on the use of inorganic gases above their critical point as mobile phase in chromatography systems stems back to 1957^1^ and it was Klesper *et al* who reported on a preliminary study in which chlorofluorocarbons were used above their critical point to separate metal phophorins.^2^ Supercritical fluid chromatography (SFC) with carbon dioxide as the mobile phase emerged in the late 1960s. In the past decade, a new generation of SFC instruments are commercially available and also the number of applications of SFC have grown considerably. Here, Susan Olesik, Caroline West, Davy Guillarme, Debby Mangelings, and Lucie Nováková share their thoughts on the technology and discuss the challenges and potential of SFC.

## 1 WHAT WAS YOUR FIRST EXPERIENCE WITH SFC?

*Susan Olesik*: I joined Milos Novotny's group in 1982. This was in the very early days of capillary Supercritical fluid chromatography. While I was in the Novotny group, we published the first SFC‐FTIR interface.

*Caroline West*: I started doing SFC as a PhD student. It was the only technique I was supposed to investigate for my thesis. I was drawn to the topic because my mentor convinced me that it was a technique worth learning, because there were still lots of unknowns. As one who likes to learn new things, this was obviously attractive to me. From a more practical point of view, it means there is still a lot to do so it may be relatively easy to find some novelty and produce some interesting research.

*Davy Guillarme*: In 2011, Waters came to our laboratory to initiate a collaboration in the field of SFC for pharmaceutical analysis. My supervisor (Prof. Jean‐Luc Veuthey) has a strong background in SFC and was involved in SFC during the 80′s, but he stopped this research line at the end of the 90′s. Therefore, he was very interested to see what can be done with SFC today. On my side, I did not have a good feeling about SFC, since I heard many negative things during my scientific career (i.e. lack of repeatability, poor quantitative performance, unsuitable robustness). In addition, I was convinced that SFC could not compete with UHPLC at that time. Anyway, we had a 2‐years loan from Waters for a Thar SFC system, since they just purchased this company. The first experiments conducted on this system at the analytical scale confirmed my negative impression. Indeed, the system looks like an old HPLC system, with large volumes everywhere, retention times and peak area repeatability was horrible, a strong carryover was observed, and we had a lot of technical issues, in particular with the back pressure regulator. So, the performance of SFC was clearly disappointing and I wanted to stop this collaboration, since I did not see any future for SFC at that time, in comparison with UHPLC.

*Debby Mangelings*: Our laboratory defined generic separation strategies that guide analysts in chiral method development for more than 10 years. This started as a cooperation with Sanofi and a variety of separation techniques was used for these strategies, such as reversed‐phase LC, normal‐phase LC, capillary electrophoresis, and capillary electrochromatography. A chiral separation strategy for SFC also was defined together with our group, but we did not have the technique in our laboratory at that time. With the introduction of a new generation of polysaccharide selectors, there was a need to update our separation strategies for all techniques. Therefore, we decided to start a project to also update the existing chiral separation strategy in SFC and we purchased our first (older generation) instrument in 2010. Immediately, I was impressed by the speed of analysis in SFC.

*Lucie Nováková*: I have to say, I am quite young to SFC community, especially compared to the pioneers of this technique, those like Terry Berger, David Pinkston, Larry Taylor, and many others. I have an admiration for these researchers who witnessed the early development of this technique and contributed to its progress. I was the “lucky one” who first got in touch with what is called advanced SFC platform. So, the instruments I have been using had the painful issues of back‐pressure regulation or stable pumping already overcame. My first experience dates back in December 2012. I had a chance of testing a new SFC platform without any previous experience with SFC, which was challenging, but attractive task. Our focus is pharmaceutical analysis in all its possible forms starting from quality control of pharmaceuticals, going through bioanalysis and plant material analysis ending up with food analysis and dietary supplements control. My curiosity of course resulted in testing those separations that were troublesome to optimize in LC and to my great pleasure and surprise, many of them were easy and straightforward in SFC. This story had an unexpected happy‐end, because this demo instrument did not leave our laboratory any more. We directly knew that we need such a technique in our lab, so we kept this first instrument and completed our SFC portfolio with two more of the same kind.

## 2 WHAT WAS YOUR BIGGEST SUCCESS IN THE FIELD OF SFC?

*Susan Olesik*: My research group was the first to develop enhanced‐fluidity liquid chromatography (EFLC) in 1991.^3^ Enhanced fluidity liquids are mixtures of common liquids with liquefied gases, such as carbon dioxide. We measured phased diagrams, diffusion coefficients of analytes in a range of mixtures, measured the polarity of the mixtures and illustrated at the time the enhanced efficiency compared to conventional HPLC. I consider EFLC to be an extension of SFC. Most of the work today in SFC is done in the subcritical portion of phase diagrams. Today, the SFC commercial instrumentation is typically designed, through software controls to not venture to proportions of cosolvent >40‐50 v/v%. EFLC starts with the equivalent of 100% cosolvent and adds CO_2_. Most recently our group illustrated that ELFC interfaced with electrospray ionization (ESI) sources, provides marked improvements in detection limits for proteins and provides the capability to shift the charge distribution for proteins to higher charge states.^4^

*Caroline West*: To have designed a classification of columns for SFC use, which has been very helpful in understanding the different modes of operation depending on the choice of stationary phase. SFC is often described as a normal‐phase technique, but I disagree with this view. This is far too restrictive to see it through this simple scope. SFC can be normal‐phase, but it can also be reversed‐phase, mixed‐mode or even ion‐exchange. It all depends on the choice of stationary phase.

*Davy Guillarme*: In 2012, Waters suggested to put their new prototype SFC system (Acquity UPC2) in our laboratory. In my opinion, this was really a revolution for SFC, since columns packed with small particles can be more easily used on this system. In addition, the robustness, sensitivity, quantitative performance and repeatability greatly improved on this instrument, compared to the previous generations of SFC systems. The changes were so obvious with the new instrument that it looks like the transition from HPLC to UHPLC in 2004. Today, Agilent and Shimadzu are also selling such modern SFC systems. One of the biggest success we had with SFC was the evaluation of this new UHPSFC system for pharmaceutical analysis, both from a fundamental point of view (kinetic evaluation) and more practical aspects (selectivity, peak shapes, retention of polar compounds…). In this context, we systematically compared the performance of regular SFC versus UHPSFC, using columns packed with different particle sizes. The first article we published on this topic in 2012 has already been cited more than 130 times, which is a very good achievement.

*Debby Mangelings*: In fact, all projects in our lab that have been completed with SFC until now were successful. The chiral project was a start‐up of our SFC experience, and a considerable number of papers was generated during this period. It also led to an updated chiral separation strategy in SFC with a very high success rate. In a follow up period, we started a project around drug impurity profiling using achiral SFC, which also resulted in a high number of publications. These two projects can be considered as the most successful ones up till now, but in fact we keep on implementing SFC in our current research, so definitely our list will become even longer.

*Lucie Nováková*: The tutorial paper on “Modern analytical supercritical fluid chromatography using columns packed with sub‐2 µm particles” co‐authored by Davy Guillarme, Alexandre Grand‐Guillaume Perrenoud, Isabelle Francois, Caroline West, and Eric Lesellier for Analytica Chimica Acta in 2014^5^ has received particular recognition. I know many people who used it to learn the basics of SFC or to enhance their understanding of the technology. With respect to experimental work, I consider our contributions in the field of implementation of SFC in pharmaceutical analysis or bioanalysis equally successful. Definitely, the most original work was the one where we first used polymer monolithic columns for SFC analysis of polypeptides, but this was a pilot work that still needs a lot of improvement. I was very happy that I could make a part of very interesting UHPSFC‐MS/MS projects focused on antidoping analyses in collaboration with University of Geneva.

## 3 HAS SFC CURRENTLY EARNED ITS PLACE IN THE ANALYTICAL LABORATORY?

*Susan Olesik*: Absolutely. With the today's packed columns that are specially designed for SFC separations, efficient and fast separations can be garnered for a large range of nonvolatile compounds. Furthermore, especially for semi‐preparative and preparative columns, SFC provides a green method for the separation and collection of compounds. This is particularly important in the pharmaceutical industry.

*Caroline West*: I guess it depends on what analytical laboratory we are talking about. For chiral separations, especially at the preparative scale, it was proven long ago as the fastest and most productive way to achieve enantioresolution. For achiral separations, much progress has been done in the past decade, which are mostly related to improved instruments and facilitated hyphenation to mass spectrometry. This was helpful in finally catching attention of many scientists, who are interested in developing new applications. However, SFC must always prove itself to convince the sceptics. I am not a fanatic of the technique, I am not saying everyone should use it. I just think it is a brilliant technique and it is a pity not to try it.

*Davy Guillarme*: For people who are familiar with modern SFC (or UHPSFC), I would say that SFC has definitively its place in the analytical laboratory. First, it is highly complementary to RPLC, since it provides orthogonal selectivity (SFC looks like NPLC in terms of retention behavior), it is also able to better retain polar compounds (due to the use of polar stationary phases in most of the cases) and is able to elute more easily highly lipophilic compounds (similarly to what can be done in NPLC). Second, it is a green technique, which uses a mobile phase mostly composed of CO_2_, with limited amount of organic solvents. In addition, SFC can be easily coupled with MS detection. Finally, it is certainly the best analytical technique for chiral separations and preparative applications. However, for people who never have the opportunity to work with modern SFC instrumentation, they still believe that SFC only has drawbacks, similarly to what I was thinking before using modern SFC devices. Therefore, these scientists are reluctant to using SFC and the lack of education on SFC is certainly one of the reasons why it is today less widely used than GC or LC in a modern analytical laboratory. However, it is a very powerful analytical technique!

*Debby Mangelings*: For chiral separations, both analytical and preparative, it is certainly an established technique in both academic and industrial labs. For achiral applications, the scientific community has an increased interest in the technique for all types of applications since the introduction of the new generation instruments, which eliminated most of the drawbacks at instrumental level. In academic labs where SFC is used, I am convinced that SFC has earned its place. However, SFC is not much used in an industrial environment for routine analysis. What should be demonstrated more in papers is the added value of SFC compared to LC, as such information is needed to convince users to also consider SFC for their applications. In addition, a better understanding of the fundamentals of SFC is still needed, as many parameters impact a separation.

*Lucie Nováková*: This is definitely the case in our laboratory. We are very happy to use the three SFC systems and they are running most of the times. We are using them for a variety of projects to answer fundamental research questions as well as for method development for high‐end applications. SFC is a powerful technique, complementary to HPLC and GC. I was impressed many times with its performance when developing chiral methods or the method for structurally very close derivatives of vitamin E or steroids. However, this is the situation in academic research laboratory. The implementation of SFC in routine laboratories, especially in those with regulated environment, will be much more difficult and will take much more time. For these laboratories SFC still remains a niche technique, unfortunately. I am definitely a strong promoter of SFC and will always be happy to help changing this situation.

## 4 WHAT ARE THE ANALYTICAL CHALLENGES CURRENTLY EXPERIENCED AND WHAT ADVANCES IN SYSTEM DESIGN AND COLUMN DESIGN ARE NEEDED?

*Susan Olesik*: Chromatographic system designs should be available to quickly and with high precision switch between HPLC, EFLC, SFC and UHPLC. There should be no need for separate instruments.

*Caroline West*: Biomolecules are an interesting challenge for SFC because large and polar molecules do not look soluble in carbon dioxide‐based mobile phases so this is not where SFC would seem like an immediate choice. But I have little interest for the obviousness, I like to explore the edges, and biomolecules are one of the last areas remaining to explore. Regarding system design, I would like bigger ovens to fit longer column lengths, because you can play with different selectivities and achieve very high plate numbers for difficult separations. It is relatively easy in SFC as the fluid has low viscosity, but we would then need higher pressure systems (currently limited to 400‐660 bars, depending on manufacturer). I'm also greatly interested in multi‐dimensional systems: 2D‐SFC but also combining different fluids (liquids and supercritical fluids for instance).

*Davy Guillarme*: In SFC, there are still a few challenges that need to be addressed. First of all, when a method has to be transferred between different instruments, various column dimensions (for example, during a transfer from analytical to preparative scale) or simply when the flow rate has to be changed, there could be a strong impact on the method selectivity and retention, leading to loss in resolution, due to the modification of the fluid density. This is something that should be better understood, and I strongly advise scientists in the field of fundamental chromatography to work on this topic and suggest a “simple” way to perform method transfer in SFC. Secondly, one of the main issues related to SFC is the large system volumes of modern SFC systems that hamper the use of columns with diameters of 1 or 2.1 mm I.D. Therefore, 3 mm I.D. columns are still the reference today in SFC to limit loss in performance due to the system. This is unfortunate since the solvent consumption is not decreased as much as it should be, and the modern SFC technique with 3 mm I.D. columns is not much greener than UHPLC with 2.1 mm I.D. columns. Last, there would also be a need to find an innovative chemistry dedicated for SFC that would not necessitate the addition of salts and water in the mobile phase, in particular when analyzing basic molecules. There are already the 2‐EP and 2‐PIC columns that exist but they do not always work. This aspect would be particularly important for preparative SFC scale applications.

*Debby Mangelings*: If one wants to couple columns for more complex separations, the pressure limit of the instrument is reached quite fast, so if this would be higher, it would certainly bring some additional possibilities for the future. There is also a need for instruments with lower extra column volumes, as these compromise the efficiency of the separations on small diameter columns. Finally, the column ovens in recently commercialized instruments are not designed for long columns, making it necessary to use an external column oven, with additional extra column volumes as a consequence.

*Lucie Nováková*: Current SFC systems can tackle lot of drawbacks related to the older platforms. However, the issues, such as still large extra‐column volumes or relatively low maximum system pressure still need substantial improvement. It will be also interesting to design fluorescence detector for SFC. This is currently impossible due to low pressure resistance of the available fluorescence flow cells. Concerning stationary phase, I am happy with the available stationary phases despite the fact that a universal stationary phase, such as C_18_ in RP‐HPLC, does not exist in SFC. In our lab we use a large spectrum of stationary phases both chiral and achiral, many of them originally designed for LC, so the selection of chemistries is wide. The advances in design of stationary phase may follow the development in LC, so we can start with core‐shell particles and end up with 3D printed columns one day in the future.

## 5 WHAT ADVICE CAN YOU GIVE EMERGING SCIENTIST WORKING IN THE FIELD OF SFC?

*Susan Olesik*: There is still much to learn about SFC and even liquid chromatography. The field of analytical chemistry needs fundamental scientists who are willing to study deeply chromatography. Dig in.

*Caroline West*: Forget what you have learnt in HPLC and keep an open mind to accept SFC as it is. If you keep comparing, you will never make full use of the technique.

*Davy Guillarme*: If you want to be innovative, do not hesitate to break the limits of SFC! There are still a lot of things to explore in SFC, including: (a) the analysis of very polar substances that are often problematic to analyze in RPLC conditions, (b) the characterization of peptides and proteins in SFC which looks impossible some years ago, but become a reality, (c) conducting large scale metabolomics studies in SFC‐MS to find some new biomarkers, as a complementary tool to LC‐MS, GC‐MS, or NMR, (d) the use of SFC in a quality control laboratory in the pharmaceutical industry, as a replacement strategy to RPLC.

*Debby Mangelings*: Consider to use SFC as separation technique whenever possible. You will be impressed by its possibilities and speed of analysis.

*Lucie Nováková*: Give it a try, stay inspired and creative. Actually, my advice would not be different from advising scientist in any other field of analytical chemistry: (1) Study literature carefully. Some young people do not realize how important this is. (2) Run experiments and don't be afraid of making mistakes. Mistakes can sometimes lead to interesting questions and may open up new challenging projects. (3) Be patient. Precious things take time.

## CONFLICT OF INTEREST

The authors declare that there is no conflict of interest.
